# Models of very-low-mass stars, brown dwarfs and exoplanets

**DOI:** 10.1098/rsta.2011.0269

**Published:** 2012-06-13

**Authors:** F. Allard, D. Homeier, B. Freytag

**Affiliations:** CRAL, UMR 5574, CNRS, Université de Lyon, École Normale Supérieure de Lyon, 46 Allée d'Italie, 69364 Lyon Cedex 07, France

**Keywords:** PHOENIX, CO5BOLD, very-low-mass stars, brown dwarfs, stars, opacities

## Abstract

Within the next few years, GAIA and several instruments aiming to image extrasolar planets will be ready. In parallel, low-mass planets are being sought around red dwarfs, which offer more favourable conditions, for both radial velocity detection and transit studies, than solar-type stars. In this paper, the authors of a model atmosphere code that has allowed the detection of water vapour in the atmosphere of hot Jupiters review recent advances in modelling the stellar to substellar transition. The revised solar oxygen abundances and cloud model allow the photometric and spectroscopic properties of this transition to be reproduced for the first time. Also presented are highlight results of a model atmosphere grid for stars, brown dwarfs and extrasolar planets.

## 1. Introduction

Since the spectroscopic observations of very-low-mass stars (VLMs, late 1980s), brown dwarfs (mid-1990s) and extrasolar planets (mid-2000s) have become available, the MK spectral classification has had to be extended beyond K and M to the newly defined classes L and T. One of the most important challenges in modelling their atmospheres and spectroscopic properties has been high-temperature molecular opacities and cloud formation. The K dwarfs show the onset of formation of metal hydrides (starting around *T*_eff_∼4500 K), TiO and CO (below *T*_eff_∼4000 K), while water vapour forms in early M dwarfs (*T*_eff_∼3900–2000 K), and methane, ammonia and carbon dioxide are detected in late-type brown dwarfs (*T*_eff_∼300–1600 K) and in extrasolar giant planets. The latter are observed either by transit (*T*_eff_∼1000–2000 K, depending on the spectral type of the central star and the distance to the star) or by imaging (young planets of *T*_eff_∼300–2000 K, depending on their mass and age).

The modelling of the atmospheres of VLMs has evolved (as illustrated here with the development of the PHOENIX atmosphere code, which has allowed the detection of water vapour in the atmospheres of extrasolar planets by Barman *et al.* [1,2]) with the extension of computing capacities from an analytical treatment of the transfer equation using moments of the radiation field [3], to a line-by-line opacity sampling in spherical symmetry [4–6] and, more recently, to three-dimensional radiation transfer [7]. In parallel with detailed radiative transfer in an assumed static environment, hydrodynamical simulations have been developed to reach a realistic representation of the granulation and its induced line shifts for the Sun and Sun-like stars [8] by using a non-grey (multi-group binning of opacities) radiative transfer with a pure blackbody source function (scattering is neglected).

## 2. Molecular opacities

**Figure 1.**
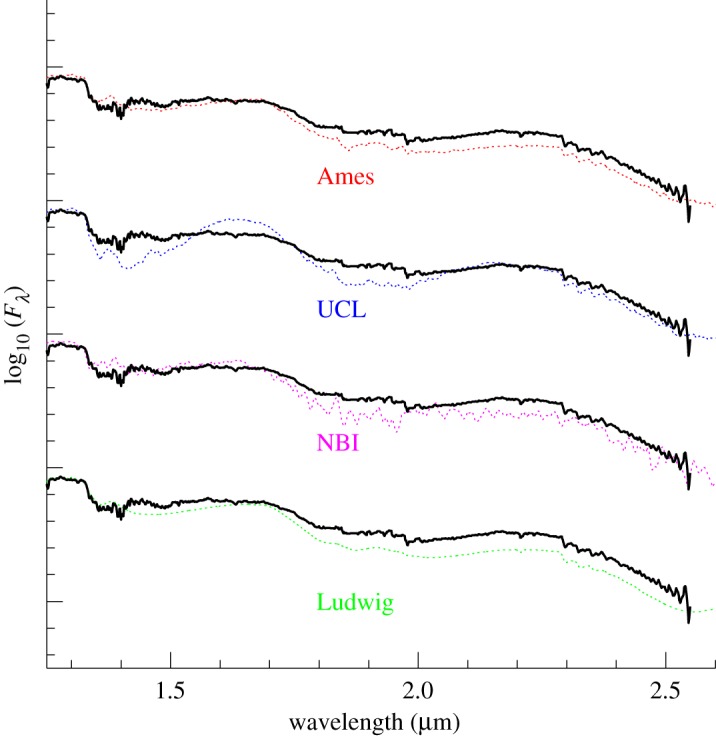
Synthetic spectra compared with the infrared spectral energy distribution of the M8e dwarf VB10 using identical model parameters (*T*_eff_=2800 K, 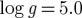) and a resolution of 50 Å with different water vapour opacity sources: the Base grid by Allard & Hauschildt [13] using the study of Ludwig [14]; a test using the 1994 version of the Niels Bohr Institute [15]; the NextGen grid by Allard *et al*. [4,16] and Hauschildt *et al*. [5,6] using the University College London database [17]; and the AMES-Cond/Dusty grid by Allard *et al*. [18,19] using the NASA-Ames Center database [20]. All models (except the NextGen/UCL case) underestimate the flux at *K* (*ca* 2.0–2.4 μm) by 0.1–0.2 dex. (Online version in colour.)

While earlier work was developed for the study of red giant stars, the pioneering work on the modelling of VLM atmospheres has been provided [3,9,10] using a band model or just overlapping line approximation opacities developed by Kivel *et al*. [11] and adapted for astrophysical use by Golden [12]. More realistic model atmospheres and synthetic spectra for VLMs, brown dwarfs and extrasolar planets have been made possible thanks to the development of accurate opacities calculated, often *ab initio*, for atmospheric layers where temperatures can reach 3000 K. The process of improvement has been especially remarkable in the case of water vapour line lists. Indeed, water vapour has seen an important evolution through the years, from band model approximations to straight means based on hot flame experiments, and then to *ab initio* computations. Nevertheless, the atmosphere models have failed to reproduce the strength of the water bands that shape the low-resolution (*R*≤300) infrared (IR) spectral energy distributions (SEDs) of M dwarfs. At the lower temperatures of brown dwarfs, methane and ammonia rival the effect of water. Therefore, the discrepancies in the model synthetic spectra were believed to be due to inaccurate or incomplete molecular opacities. In particular, water vapour was suspected because the discrepancies were observed at IR wavelengths in the relative brightnesses of the flux peaks between water vapour bands. As can be seen from figure 1, where the models are compared with the IR spectrum of the M8e dwarf VB10, the water vapour opacity profile that shapes this part of the spectrum has changed strongly over time with the improvement of computational capacities and better knowledge of the interaction potential surface. The most recent *ab initio* results confirm the earliest hot flame laboratory experimental results by Ludwig [14]. However, in general, most opacity profiles produce an excess opacity (or lack of flux in the model) in the *K* bandpass. Only the UCL1994 line list (owing to incompleteness, and with many of its deviations cancelling out over the bandpasses) could produce seemingly correct *J*−*K*_s_ colours.

## 3. The revised solar abundances

**Figure 2.**
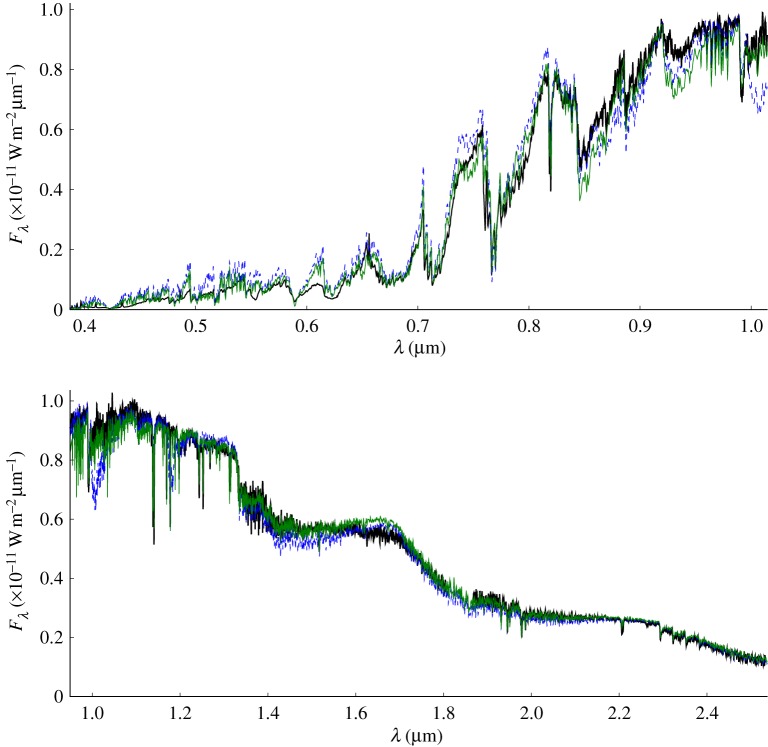
A BT-Settl synthetic spectrum with 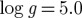 and solar metallicity by Asplund *et al*. [21] (thin grey/green full line; [M/H]=0.0) compared with the combined SED of the red dwarf triple system Gl 866 [24,25]. The observations of GJ 866ABC were combined from a Mt. Stromlo optical spectrum (M. Bessell 2009, private communication) and SpeX IR spectrum taken at the NASA IRTF [26] (thick black curve). For comparison a model using the same parameters and physical setup with the Grevesse *et al.* [23] abundances is also shown (grey/blue dashed line). The models have been scaled to the observed absolute flux assuming two equal *T*_eff_=2920 K components of 0.157 *R*_⊙_ (solar radii) and a third with *T*_eff_=2700 K and 0.126 *R*_⊙_. (Online version in colour.)

**Figure 3.**
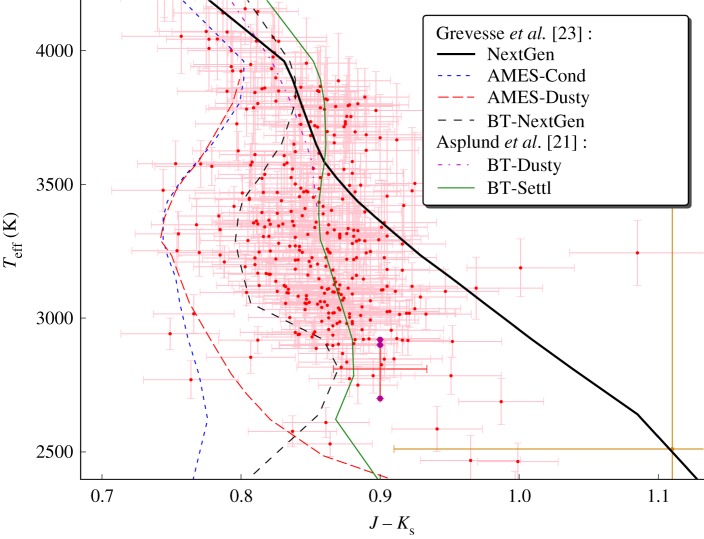
The estimated *T*_eff_ for M dwarfs by Casagrande *et al*. [27] and for brown dwarfs by Golimowski *et al*. [28] and Vrba *et al*. [29] are compared with the NextGen isochrones for 5 Gyr [30,31] using various generations of model atmospheres: NextGen (thick black line), the limiting AMES-Cond/Dusty cases by Allard *et al*. [19] (dotted blue and dashed red lines), and the current BT-Settl models using the Asplund *et al.* [21] solar abundances (full green line). The Gl 866 system fitted in figure 2 is highlighted by darker colours and shown with its relatively large photometric error bars at *J*−*K*_s_=0.9. (Online version in colour.)

Model atmospheres for VLMs and, in general, for other stars assume scaled solar abundances for all heavy elements, with some enrichment of α-process elements (the result of ‘pollution’ of the star-forming gas by the explosion of a supernova) when appropriate in the case of metal-depleted subdwarfs of the Galactic thick disc, halo and globular clusters. The revision of the solar abundances based on radiation hydrodynamical (RHD) simulations of the solar atmosphere, on improvements in the quality of the spectroscopic observations of the Sun, and on its detailed line profile analysis by two separate groups using independent hydro codes and spectral synthesis codes [21,22] yield an oxygen reduction of 0.11–0.19 dex (up to 34%) compared with the previously used abundances of Grevesse *et al.* [23]. Since the overall SED of late K dwarfs, M dwarfs, brown dwarfs and exoplanets is governed by oxygen compounds (TiO and VO in the optical, and water vapour and CO in the IR), the input elemental oxygen abundance used in the equation of state is of major importance. Figure 2 shows an example of these effects for the optical and IR SED of the M5.5 dwarf system Gl 866. However, at other effective temperatures, even stronger photometric effects can be seen, where the near-IR SEDs of different models diverge more (figure 3). The comparison shows significant improvement when compared with the older models shown in figure 1, except for excess flux in the *H* bandpass near 1.7 μm due to incomplete FeH opacity data for this region. The comparison has particularly improved in the Wing Ford band of FeH near 0.99 μm, and in the VO bands thanks to line lists provided by B. Plez (GRAAL, Montpellier, France), although inaccurate or incomplete opacities still affect the models at optical wavelengths (e.g. the TiO line list by Langhoff [32]).

Figure 3 compares the theoretical isochrones (assuming an age of 5 Gyr) with the *T*_eff_ estimates [27] and reveals that the NextGen models [4–6] systematically and increasingly overestimate *T*_eff_ through the lower main sequence, while the AMES-Cond/Dusty [19] models, on the contrary, underestimate *T*_eff_ as a function of *J*−*K*_s_ colour. This situation is relieved when using the current models (labelled BT-Settl in the figure) based on the revised solar abundances, and the models now agree fairly well with most of the empirical estimations of *T*_eff_. The current model atmospheres have not yet been used as surface boundary conditions to interior and evolution calculations, and simply provide the synthetic colour tables interpolated on the published theoretical isochrones [31]. Even if the atmospheres partly control the cooling and evolution of M dwarfs [33], differences introduced in the surface boundary conditions by changes in the model atmosphere composition have a negligible effect.

## 4. Cloud formation

One of the most important challenges in modelling these atmospheres (below 2600 K) is the formation of clouds. Tsuji *et al.* [34] identified dust formation by recognizing the condensation temperatures of hot dust grains (enstatite MgSiO_3_, forsterite Mg_2_SiO_4_ and corundum Al_2_O_3_ crystals) that occur in the line-forming layers (*τ*≈10^−4^ to 10^−2^) of their atmospheres. The cloud composition, according to equilibrium chemistry, goes from zirconium oxide (ZrO_2_), to refractory ceramics (perovskite CaTiO_3_ and corundum Al_2_O_3_), to silicates (e.g. forsterite Mg_2_SiO_4_), to salts (CsCl, RbCl, NaCl) and finally to ices (H_2_O, NH_3_, NH_4_SH) as brown dwarfs cool down over time from M through L, T and Y spectral types [19,35]. This assumed (by Allard *et al.* [19]) that sub-micrometre-sized crystal formation causes the weakening and vanishing of TiO and VO molecular bands (via CaTiO_3_, TiO_2_ and VO_2_ grains) from the optical spectra of late M and L dwarfs, revealing CrH and FeH bands otherwise hidden by the molecular pseudo-continuum, and the resonance doublets of alkali transitions, which only condense onto salts in late T dwarfs. The scattering effect of this fine dust is Rayleigh scattering, which provides veiling to the optical SED of late M and L dwarfs, while the greenhouse effect due to the dust cloud causes their IR colours to become extremely red when compared with those of hotter low-mass stars. The upper atmosphere, above the cloud layers, is depleted of condensible material and significantly cooled down by the reduced or missing pseudo-continuum opacities.

**Figure 4.**
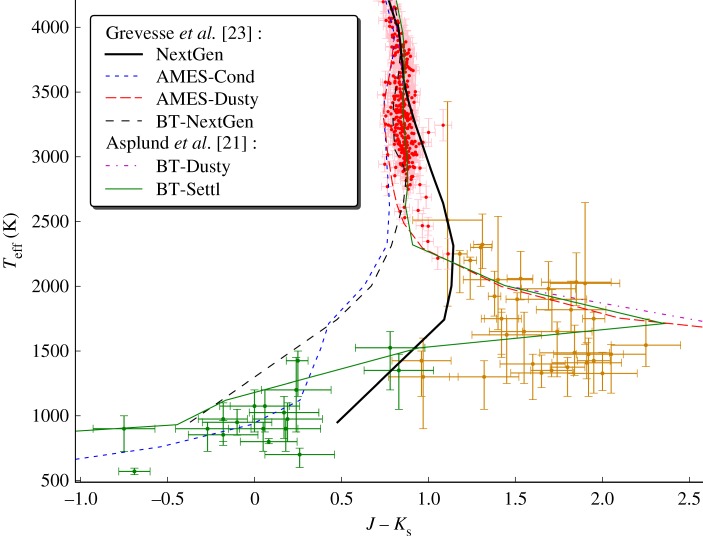
Same plot as figure 3 but zooming out and extending into the brown dwarf region of the diagram. This region below 2500 K is dominated by dust formation (essentially forsterite and other silicates). The AMES-Cond/Dusty model atmosphere limiting cases provide a description of the span in colours of the brown dwarfs in this diagram for a given age (here 5 Gyr). The BT-Settl models succeed in explaining even the most extreme colours of brown dwarfs. (Online version in colour.)

One common approach has been to explore the limiting properties of cloud formation. One limit is the case where sedimentation or gravitational settling is assumed to be fully efficient, such as case B of Tsuji [36], the AMES-Cond or condensed phase models of Allard *et al.* [19], the clear case of Ackerman & Marley [37] and the cloud-free case of Burrows *et al.* [38]. The other limit is the case where gravitational settling is assumed to be inefficient and dust, often only forsterite, forms in equilibrium with the gas phase, such as case A of Tsuji [36], the AMES-Dusty or dusty models of Allard *et al.* [19], the cloudy case of Ackerman & Marley [37] or case B of Burrows *et al.* [38]. These limiting cases of maximum dust content agree in describing the evolution of brown dwarfs from a molecular opacity-governed SED towards a blackbody SED below 1500 K. This description was suitable, at least in the case of the AMES-Dusty models, in reproducing the IR colours of L dwarfs. The cloud-free limiting case, on the other hand, allowed the colours of T dwarfs to be reproduced to some degree. Figure 4 shows this situation for the AMES-Cond/Dusty limiting case models of Allard *et al.* [19] compared with the effective temperature estimates obtained by integration of the observed SED [28,29].

The purpose of a cloud model is therefore to go beyond these limiting cases and define the number density and size distribution of condensates as functions of depth in the atmosphere. The discovery of dust clouds in M dwarfs and brown dwarfs has therefore triggered the development of cloud models building upon the pioneering work in the context of planetary atmospheres developed in the earlier studies [39–41]. The Lewis model is an updraft model (considering that condensation occurs in a gas bubble that is advected from deeper layers). Owing to lack of knowledge of the velocity field and diffusion coefficient of condensates in the atmospheres of the planets of the Solar System, Lewis [39] simply assumed that the advection velocity is equal to the sedimentation velocity, thereby preserving condensible material in the condensation layers. This cloud model did not account for grain sizes. Rossow [40], on the other hand, developed characteristic time scales as a function of particle size for the main microphysical processes involved (condensation, coagulation, coalescence and sedimentation). The intersections of these characteristic time scales give an estimate of the condensate number densities and mean grain sizes. However, this model made several explicit assumptions concerning the efficiency of supersaturation, coagulation, etc.

Helling *et al.* [42] have compared different cloud models and their impact on model atmospheres. Most cloud models define the cloud base as the evaporation layer provided by the equilibrium chemistry. In the unified cloud models of Tsuji and co-workers [36,43], a parametrization of the radial location of the cloud top by way of an adjustable parameter *T*_crit_ was used. This choice permits the cloud extension effects on the spectra of these objects to be determined but does not allow the stellar–substellar transition to be reproduced with a unique value of *T*_crit_, as the cloud extension depends on the atmospheric parameters.

Allard *et al.* [44], using PHOENIX and the index of refraction of up to 40 condensible species, have applied the Rossow cloud model, ignoring coalescence and coagulation, and comparing the time scales of condensation, sedimentation and mixing (extrapolated from the convective velocities into the convectively stable layers), and assuming efficient nucleation (monomer equilibrium densities). The cloud model was then solved layer by layer inside out to account for the sequence of formation of grain species as a function of cooling of the gas. However, this version of the BT-Settl (with gravitational settling) models did not allow the formation of enough dust in brown dwarf atmospheres, owing to a very conservative prescribed supersaturation value.

Ackerman & Marley [37] have solved the particle diffusion problem of condensates by assuming a parametrized sedimentation efficiency *f*_sed_ (constant through the atmosphere) and a mixing assumed constant and fixed to its maximum value (maximum of the inner convection zone). Saumon & Marley [45] found that their models could not produce the colour change with a single value of *f*_sed_.

Helling *et al.* [46] used the PHOENIX code to compute the Drift–Phoenix models. The cloud model used, in contrast to all other cases mentioned, studies the nucleation and growth of grains as they sediment down into the atmosphere. This cloud model determines the number density and size distribution of grains by one-dimensional nucleation simulations, and the resulting distribution is read in by PHOENIX, which computes the resulting opacities and radiative transfer. These models solve the nucleation problem, but only for the assumed monomer types, and have been successfully applied to fit the dusty atmospheres of L dwarfs, but the reversal in IR colours observed for the L–T transition could not be explained [47].

However, none of these models treated the mixing properties of the atmosphere and the resulting diffusion mechanism realistically enough to reproduce the brown dwarf spectral transition without changing the cloud parameters. Freytag *et al.* [48] have therefore addressed the complementary though important issue of mixing and diffusion in these atmospheres by two-dimensional RHD simulations, using the PHOENIX gas opacities in a multi-group opacity scheme and forsterite with geometric cross sections. These simulations assume efficient nucleation, using monomer densities estimated from the total available density of silicon (least abundant element in the solar composition involved in forsterite). They found that gravity waves play a decisive role in cloud formation, while around *T*_eff_≤2200 K the cloud layers become optically thick enough to initiate cloud convection, which participates in the mixing. Overshoot can also be important in the deepest layers.

These RHD simulations allow an estimation of the diffusion processes that bring fresh condensible material from the hotter lower layers to the cloud-forming layers. We have therefore updated our cloud model (BT-Settl models) to account for the mixing prescribed by the RHD simulations. Another important improvement concerns the supersaturation, which has been computed rather than using the fixed conservative value recommended by Rossow. One can see from figure 4 that the late-type M and early-type L dwarfs behave as if dust is formed nearly in equilibrium with the gas phase, with extremely red colours in some agreement with the BT-Dusty models. The BT-Settl models reproduce the main sequence down to the L-type brown dwarf regime, subjected in the *K* bandpass to the greenhouse effect of dust clouds, before turning to the blue in the late L and T dwarf regime as a result of methane formation in the *K* bandpass. This constitutes a major improvement over previous models, and shows promise that, in the near future, we can reach a clear explanation of the stellar–substellar transition.

Diffusion has also been held responsible for deviations in ultracool atmospheres from gas-phase chemical equilibrium, as noted in early observations of T dwarfs showing an excess of carbon monoxide absorption [49,50]. More recently, carbon dioxide [51], which was not expected at such low temperatures, has been detected. Similarly, ammonia has been shown to be underabundant [52]. This is understood as the result of slowing down of crucial chemical reaction steps, so that some important molecules (CH_4_, NH_3_) would not have the time to form in equilibrium while undergoing mixing, whereas others (CO, CO_2_, N_2_) remain at enhanced abundances. The RHD simulations of Freytag *et al.* [48] have allowed the underlying mixing processes to be understood, obviating the need to describe them with an additional free parameter.

## 5. Applications to exoplanet science

Several IR integral field spectrographs combined with coronograph and adaptive optic instruments that are being developed will come online before 2013 (SPHERE at the Very Large Telescope (VLT), the Gemini Planet Imager at Gemini South, Project1640 at Mount Palomar, etc.). The 39 m European Extremely Large Telescope (E-ELT) at Cerro Armazones in Chile due around 2020 will also be very ideally suited for planet imaging. The models developed for VLMs and brown dwarfs are a unique opportunity, if they can explain the stellar–substellar transition, to provide great support for the characterization of imaged exoplanets. We have therefore developed the BT-Settl model atmosphere grid to encompass the parameter regime of these objects (surface gravity around 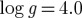, *T*_eff_<2000 K).

These planets are typically found at several dozens of astronomical units (AU) from the star, and, since the observations are done in the IR, the non-irradiated models can even be used directly. Indeed, Barman *et al.* [53] have shown that the effects of radiation from a star impinging on the planetary atmosphere are Rayleigh scattering of the stellar light by H_2_ molecules (or clouds, if present) at optical wavelengths (below 1 μm for solar-type stars), while the impact on the interior and evolution properties becomes negligible for orbital distances exceeding 0.1 AU. Nevertheless, for 2012 we are developing irradiated models and the capacity to compute them via the PHOENIX simulator (see §6).

## 6. Summary and future prospects

We report progress on the development of a new model atmosphere grid for stars, brown dwarfs and young planets, named BT-Settl. It has been computed using the PHOENIX code updated for: (i) the line lists of water by Barber & Tennyson (BT2) [54], methane using the Spherical Top Data System (STDS) [55], ammonia [56] and CO_2_ opacity from the Carbon Dioxide Spectroscopic Databank (CDSD-1000) [57], (ii) the solar abundances revised by Asplund *et al*. [21] and (iii) a cloud model accounting for more detailed supersaturation and RHD mixing. The grid covers the whole range of stars to young planets, 400 *K*<*T*_eff_<70 000 *K*, 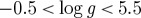 and −4.0<[M/H]<+0.5, including values of the α-element enhancement (supernovae enrichment of the star-forming material) between +0.0 and +0.6. Models are available at the PHOENIX simulator website (http://phoenix.ens-lyon.fr/simulator/) and are in preparation for publication to serve, among others, the GAIA, MUSE and SPHERE/GPI/P1640 instruments due to come online in the near future. Corresponding evolution models are expected for 2012.

We found that the previously used NextGen models systematically overestimate *T*_eff_ below 3500 K by as much as 500 K. The water vapour opacity profile has converged with the most recent line lists reproducing laboratory results, but could not explain this discrepancy. The solution came instead from the revision of the solar abundances, which changes the strength of the water vapour absorption bands, and therefore allows the reproduction of the spectroscopic and photometric properties of M dwarfs as late as M6. Later-type M dwarfs are affected by dust formation, and cloud modelling is important to understand their properties. We find that the Rossow cloud model allows, with revisions to the supersaturation and mixing, the stellar–substellar transition to be reproduced. A small offset persists, however, in the M–L transition. It is possible that all the current cloud models are not efficient enough in producing dust at the onset of the cloud formation regime. Detailed nucleation studies could allow this issue to be resolved in the future. Other uncertainties affect the current cloud modelling, such as the assumption of spherical non-porous grains, whereas grains form as fractals in the laboratory. Constraining the models therefore remains very important.

Beyond cloud modelling and molecular opacities, model atmospheres for these objects require reaction rates for the most abundant molecules and/or most important absorbers. Furthermore, these atmospheres are composed of molecular hydrogen, which constitutes the main source of collisions. Also needed therefore are collision rates (by H_2_) and corresponding damping constants for the broadening molecular lines.

In order to say something about the spectral variability of VLMs, brown dwarfs and planets, three-dimensional global or ‘star-in-a-box’ RHD simulations with rotation will be required. This is our current project supported by the French ‘Agence Nationale de la Recherche’ for the period 2010–2015.
